# Establishment of Bovine-Induced Pluripotent Stem Cells

**DOI:** 10.3390/ijms221910489

**Published:** 2021-09-28

**Authors:** Yue Su, Ling Wang, Zhiqiang Fan, Ying Liu, Jiaqi Zhu, Deborah Kaback, Julia Oudiz, Tayler Patrick, Siu Pok Yee, Xiuchun (Cindy) Tian, Irina Polejaeva, Young Tang

**Affiliations:** 1 Department of Animal Science, Institute of Systems Genetics, University of Connecticut, Storrs, CT 06268, USA; yue.3.su@uconn.edu (Y.S.); ling.wang@uconn.edu (L.W.); jiaqi.zhu@uconn.edu (J.Z.); julia.oudiz@uconn.edu (J.O.); xiuchun.tian@uconn.edu (X.T.); 2 Department of Animal, Dairy and Veterinary Sciences, Utah State University, Logan, UT 84322, USA; zhiqiang.fan@usu.edu (Z.F.); ying.liu@usu.edu (Y.L.); tayler.patrick@usu.edu (T.P.); 3 Department of Cell Biology, University of Connecticut Health Center, Farmington, CT 06030, USA; deborah.kaback@uchc.edu (D.K.); syee@uchc.edu (S.P.Y.)

**Keywords:** induced pluripotent stem cells (iPSCs), bovine, reprogramming, pluripotency, differentiation, embryo aggregation, nuclear transfer (NT)

## Abstract

Pluripotent stem cells (PSCs) have been successfully developed in many species. However, the establishment of bovine-induced pluripotent stem cells (biPSCs) has been challenging. Here we report the generation of biPSCs from bovine mesenchymal stem cells (bMSCs) by overexpression of lysine-specific demethylase 4A (KDM4A) and the other reprogramming factors OCT4, SOX2, KLF4, cMYC, LIN28, and NANOG (K_d_OSKMLN). These biPSCs exhibited silenced transgene expression at passage 10, and had prolonged self-renewal capacity for over 70 passages. The biPSCs have flat, primed-like PSC colony morphology in combined media of knockout serum replacement (KSR) and mTeSR, but switched to dome-shaped, naïve-like PSC colony morphology in mTeSR medium and 2i/LIF with single cell colonization capacity. These cells have comparable proliferation rate to the reported primed- or naïve-state human PSCs, with three-germ layer differentiation capacity and normal karyotype. Transcriptome analysis revealed a high similarity of biPSCs to reported bovine embryonic stem cells (ESCs) and embryos. The naïve-like biPSCs can be incorporated into mouse embryos, with the extended capacity of integration into extra-embryonic tissues. Finally, at least 24.5% cloning efficiency could be obtained in nuclear transfer (NT) experiment using late passage biPSCs as nuclear donors. Our report represents a significant advance in the establishment of bovine PSCs.

## 1. Introduction

Since 2006, induced pluripotent stem cells (iPSCs) rapidly emerged as another type of pluripotent stem cells (PSCs), which were reprogrammed from somatic cells by exogenous expression of OCT4 (also known as POU5F1), SOX2, KLF4, and c-MYC (OSKM), or OCT4, SOX2 (OS) plus LIN28 and NANOG (LN) [1,2,3]. There have been numerous efforts in the derivation of bovine pluripotent stem cells (PSCs) including bovine embryonic stem cells (bESCs) and induced pluripotent stem cells (biPSCs). Attempts to derive bESCs from the inner cell mass (ICM) of bovine embryos started more than 22 years ago [4]. However, many of the early described bESCs quickly differentiated within a few passages [5,6,7]. Recently, bovine ESC-like cells capable of long-term culture were reported [8]. These bESCs showed the expression of pluripotent genes and an epigenetic landscape similar to the human primed-pluripotent state ESCs (basic fibroblast growth factor (bFGF) dependent).

Bovine iPSCs were also reportedly induced from bovine fetal or adult cells using ectopic expression of OSKM, combined with LN (OSKMLN) or LN plus Large T antigen since 2011 [9,10,11,12,13,14,15]. These biPSC generally resembled primed-pluripotent human iPSCs and had a flat, monolayer cell colony morphology [9,10,11]. Naïve-like biPSCs resembling naïve-pluripotent mouse PSCs with dome-shaped, three-dimensional colony morphology were also reportedly reprogrammed from bovine fibroblasts, testicular cells, or amnion cells [15,16,17,18,19]. However, like their bESC counterparts, all these cells in general suffer limited self-renewal capacity. Also, most of the biPSCs, including those reported recently [19,20] still relied on the expression of exogenous transgenes for continuous propagation. Meanwhile, bovine trophectoderm cells were reported when reprogramed using conditions for biPSCs generation [15,21]. The problems of persistent transgene activity [22], limited propagation capacity of the biPSCs [20], and the unexpected generation of trophectoderm-lineage stem cells [23] indicate that the majority of reported biPSCs were partially reprogrammed. Overall, the lack of completely reprogrammed biPSCs signifies the existence of unidentified reprogramming hurdles in bovine somatic cells that prevent the generation of *bona fide* biPSCs [24]. Furthermore, PSCs are considered ideal nuclear donors after genetic-modification for nuclear transfer (NT) because of their infinite self-renewal potential. Bovine ESCs as donors for NT revealed a bovine blastocyst efficiency of 10–20% [8] or 21.2% [25], which is either lower than using the primary bovine fibroblasts as donor (29%) [8], or comparable to that (17%) [25], depending on different experimental systems and control cells used. However, so far, the NT blastocyst efficiency using biPSCs as nuclear donor has not been evaluated.

As the most common type of large domesticated ungulates, bovine contributes to 45% of the global animal protein supply for human consumption [26]. The establishment of *bona fide* biPSCs will have huge impact on agricultural and biotechnological applications, to help establish a sustainable agriculture system to accommodate the need of an increasing global population. This technology is expected to produce abundant and renewable PSC resources in laboratory settings, to better understand embryogenesis in ruminants, to help generate genetically superior cattle with improved animal health, production and reproduction via genetic screening and manipulation [8,27], and also to promote the preclinical development of stem cell-based therapeutics using bovine disease models such as citrullinemia and leukocyte adhesion deficiency [24,28,29]. However, compared with the PSCs derived from rodents and humans, the reported biPSCs still exhibit the above described major issues, which hinder their further applications downstream.

We previously reported that a combined OSKMLN plus histone-methyltransferase inhibitor iDOT1L significantly stimulated the human iPSC induction efficiency. The addition of WNT signal inhibition by IWR1 at middle-reprogramming stage further increased the completely reprogrammed cell population [30]. In this study, we report the induction of biPSC using a similar approach, with OSKMLN plus the additional expression of lysine-specific demethylase 4A (KDM4A) for reprogramming (K_d_OSKMLN), and their pluripotency characterization. We also found that using these biPSCs as nuclear donors gave a comparable blastocyst development to control adult fibroblasts.

## 2. Results

### 2.1. Establishment of Bovine iPSCs

**Figure 1:**
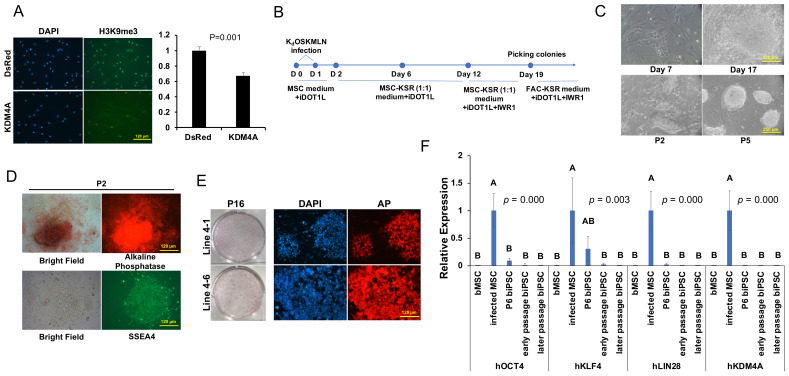
Induction of biPSCs. (**A**): Left Panel: Immunostaining of H3K9me3 expression in bMSCs infected with retroviral vector control or KDM4A. Bar = 120 µm. Right Panel: Relative fluorescence intensity for H3K9me3 in bMSCs. Bar = mean ± sd, *n* = 3. Student’s t-test was used for data analysis. (**B**): Scheme of reprogramming of the bovine MSCs. (**C**): Upper panel: Development of iPSC colonies at day 7 and 17 of retroviral K_d_OSKMLN infection of bMSCs, bar = 120 µm. Lower panel: Picked biPSC colonies at P2 and P5, bar = 250 µm. (**D**): Expression of AP (upper panel) and SSEA4 (lower panel) in P2 biPSC colonies, bar = 120 µm. (**E**): AP staining of biPSCs expanded in KT medium at P16. Left: Two AP-stained biPSC lines from one well of a 6-well plate. Right: AP-fluorescence from 2 biPSC lines under the microscope. Bar = 120 µm. (**F**): qRT-PCR for transgene expression in four lines of biPSCs at passage 6 (P6), early passages (Lines 4-1 at P17, 4-6 at P17, 3-2 at P10), and later passages (Lines 4-1 at P43, 4-6 at P25, 3-2 at P29, O4 at P40). Transgene infected bMSCs at 48 h were used as the positive control. Bar = mean ± sd, *n* = 4. One way–ANOVA with Tukey’s post hoc multiple comparison test was used for data analysis.

We previously found [30] that the combined expression of human reprogramming factors OKMSLN (expressed in three different pMXs-retroviral vectors for O, KMS, and LN), together with the inhibitor of histone methyltransferase DOT1L (iDOT1L) and the WNT inhibitor (IWR1) promoted human iPSC induction efficiency by more than 100-fold. We therefore applied the same iPSC induction scheme to reprogram the bovine primary mesenchymal stem cell-like cells (bMSCs) derived from bovine placenta (Figure S1A,B). Although we could see obvious cell aggregation in the first two weeks of reprogramming, we failed to identify the development of any PSC-like colonies thereafter. We further tried to reprogram bovine bMSCs in different PSC culture media, including the knockout serum replacement (KSR)-based ESC medium, mTeSR medium [31], and a modified epiblast stem cell (EpiSC) medium containing bFGF, Activin A, and WNT agonist CHIR99021 (FAC) [32,33], but still failed to secure any PSC-like colonies. Exotic expression of the histine lysine tri-methlation (H3K9me3) demethylase KDM4A has been reported to significantly improve blastocyst development in mouse and human NT experiments [34]. Also, overexpression of the H3K9me3 demethylase or suppression of its methyltransferases in reprogramming improved mouse and human iPSC induction efficiency [35,36,37]. We further verified that bMSCs overexpressing human KDM4A had reduced H3K9me3 but not H3K9me2 by immunostaining (Figure 1A and Figure S1C). Based on these lines of evidence, we incorporated pMXs-KDM4A in our OSKMLN reprogramming (termed here K_d_OSKMLN) (Figure 1B). We also added Z-VAD-FMK, a pan caspase inhibitor to reduce the apoptosis of bMSC caused by retroviral infection (Figure 1B). With the K_d_OSKMLN induction, we were able to observe PSC-like colonies from the reprogrammed bMSCs on day 17 (Figure 1C). The colonies were picked from day 19–30 and transferred to mitomycin C-treated mouse embryonic fibroblasts (MEF) feeders. They were cultured in a 1:1 combination of the FAC/KSR media (Figure 1B,C) for the first three passages and thereafter in 1:1 combination of KSR/mTeSR media with the addition of iDOT1L and IWR1 (termed KT medium) (Figure 1B). These bovine cells exhibited strong positive staining of the PSC surface markers alkaline-phosphatase (AP) and the stage-specific embryonic antigen 4 (SSEA4) at different passages (Figure 1D,E).

The silence of exogenous transgenes is one of the key markers for successful reprogramming [1,38,39]. We tested four lines of the established biPSCs (Lines 4-1, 4-6, 3-2, and O4) for the expression of viral transgenes. Since the K_d_OSKMLN transgenes were expressed either alone (O and Kd) or polycistronically (KMS and LN), specific PCR primers were designed to amplify DNA regions spanning the vector and the cloned human genes. qRT-PCR revealed that for all vectors, the expression of transgenes was inactivated in early passages (P10–P17) and remained silenced in later passages (P25–P43) (Figure 1F).

### 2.2. Characterization of Primed-like Bovine iPSCs

**Figure 2:**
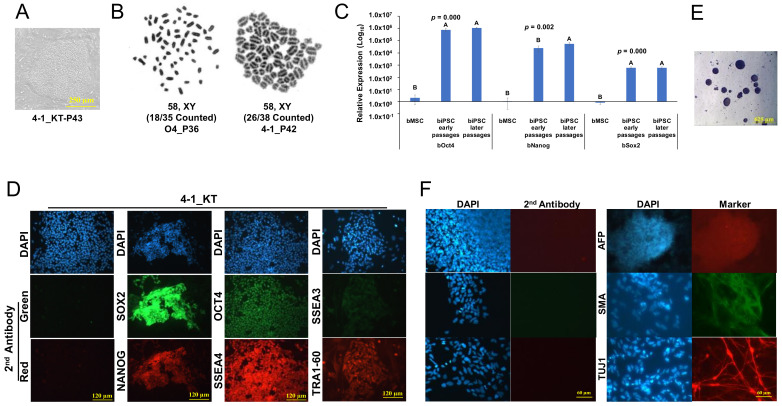
Characterization of primed-like bovine iPSCs. (**A**): Flat colony morphology of 4-1 in KT medium. Bar = 250 µm. (**B**): Karyotype for O4 and 4-1 biPSC lines at passage 36 and 42, respectively. (**C**): qRT-PCR for endogenous expression of pluripotent genes in four lines of biPSCs at early passages (4-1 at P17, 4-6 at P17, 3-2 at P10) and later passages (4-1 at P43, 4-6 at P25, 3-2 at P29, O4 at P40). Bar = mean ± sd, *n* = 4. One way–ANOVA with Tukey’s post hoc multiple comparison test was used for data analysis. (**D**): Representative immunostaining images of biPSCs in KT medium for OCT4, NANOG, SOX2, SSEA3, SSEA4, TRA-1-60. Bar = 120 µm. (**E**): EBs formed from 4-1_KT, bar = 625 µm. (**F**): Immunostaining of differentiated cells for the three-germ layer markers (AFP for endoderm, SMA for mesoderm, and TUJ1 for ectoderm) after passaging of the EBs. bar = 120 µm.

The biPSCs cultured in KT medium could be passaged continuously with collagenase treatment, and displayed the monolayered, flat, and primed-PSC colony morphology (Figure 2A). They exhibited normal karyotype (Figure 2B). We further evaluated the activation of endogenous pluripotent genes in these biPSCs. qRT-PCR of four biPSC lines using specific primers for endogenous key pluripotent genes revealed that bovine OCT4, NANOG, and SOX2 were highly activated in these biPSCs across different passages (Figure 2C). Immunostaining using specific antibodies further confirmed the expression of these pluripotent proteins in biPSCs, together with the expression of additional pluripotent surface markers including SSEA4 and TRA-1-60, and a weak but distinguishable SSEA3 (Figure 2D). The biPSCs also formed embryoid bodies (EBs) upon removal of the bFGF and culture in serum-containing medium (Figure 2E), and differentiated into cell types expressing the three-germ layer specific markers (Figure 2F).

### 2.3. Development and Characterization of Naïve-Like Bovine iPSCs

**Figure 3:**
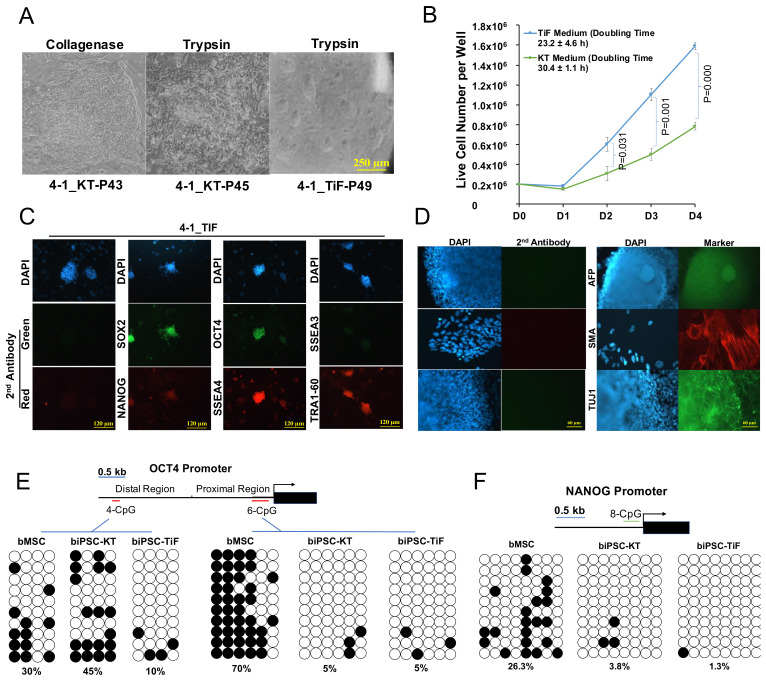
Characterization of naïve-like bovine iPSCs. (**A**): Flat, monolayered primed-like 4-1 biPSC colonies cultured in KT medium and passaged with collagenase (left), deteriorated colony morphology upon trypsinization (middle), and dome-shaped, naïve-like biPSCs cultured in TiF medium and passaged with trypsin (right), bar = 250 µm. (**B**): Cell proliferation difference between naïve and primed-like biPSCs. Mean ± sd, *n* = 3. Student’s t-test was used for data comparison. (**C**): immunostaining of naïve-like 4-1 biPSCs in TiF medium with OCT4, NANOG, SOX2, SSEA3, SSEA4, TRA-1-60 antibodies. Bar = 120 µm. (**D**): Immunostaining of EBs derived from naïve-like biPSCs for three-germ layer markers (AFP for endoderm, SMA for mesoderm, and TUJ1 for ectoderm) after passaging of the EBs, bar = 120 µm. (**E**): Bisulfite sequencing results of bovine OCT4 distal enhancer (Left Panel) and proximal promoter (Right Panel) genomic regions. Open and closed circles represent unmethylated and methylated CpGs, respectively. The percentage of methylated CpG is shown at the bottom of each sample. (**F**): Bisulfite sequencing results of bovine NANOG promoter genomic region. Open and closed circles represent unmethylated and methylated CpGs, respectively. The percentage of methylated CpG is shown at the bottom of each sample.

We noticed that similar to the primed-state PSCs reported in other species [40,41,42,43], the primed-like biPSCs cultured in KT medium could not sustain single cell colonization and are refractory to trypsinization, which resulted in deterioration of colony morphology upon passaging (Figure 3A). We then asked if these primed-like biPSCs could be converted to naïve-like PSCs in the medium containing extracellular signal-regulated kinases (ERK)1/2 and glycogen synthase kinase (GSK) 3 inhibitors (PD0325901 and CHIR99021) plus leukemia inhibitory factor (2i/LIF) [44,45]. We found that after switching the KT medium into mTeSR medium supplemented with iDOT1L, IWR1, 2i/LIF, and the adenyl cyclase activator forskolin (turned here TiF medium), the round, dome-shaped naive-like PSC colonies appeared over the next couple of passages (Figure 3A). These cells were capable of single cell colonization by trypsinization, a typical characteristic of naïve-state PSCs and valuable for genetic manipulation [46,47]. Cell proliferation assay revealed that the naïve-like biPSCs grew significantly faster than the primed-like cells, with a cell doubling time of 23.2 h compared to the 30.4 h for the primed-like biPSCs (Figure 3B). These are similar to the previously reported doubling time of naïve (~24 h) and primed (~30 h) human PSCs, respectively [48,49]. The naïve-like biPSCs expressed pluripotent gene OCT4, SOX2, and NANOG, as well as pluripotent surface markers SSEA4 and TRA-1-60 similar to biPSCs cultured in KT medium (Figure 3C). They formed EBs upon differentiation in serum-containing medium with cells expressing three-germ layer specific markers (Figure 3D). Bisulfite sequencing to the bovine genomic DNA revealed highly demethylated bovine OCT4 (Figure 3E, Right Panel) and NANOG (Figure 3F) proximal promoter region in both naïve-like and primed-like biPSCs in contrast with the highly methylated bMSCs. Human and mouse naïve-state PSCs preferably activate the OCT4 distal enhancer than proximal prompter [50,51,52,53,54,55]. Notably, the distal enhancer region of OCT4 in naïve-like biPSCs is less methylated than bMSCs and primed-like biPSCs (Figure 3E, Left Panel).

### 2.4. Global Transcriptome Analysis of Bovine iPSCs

**Figure 4:**
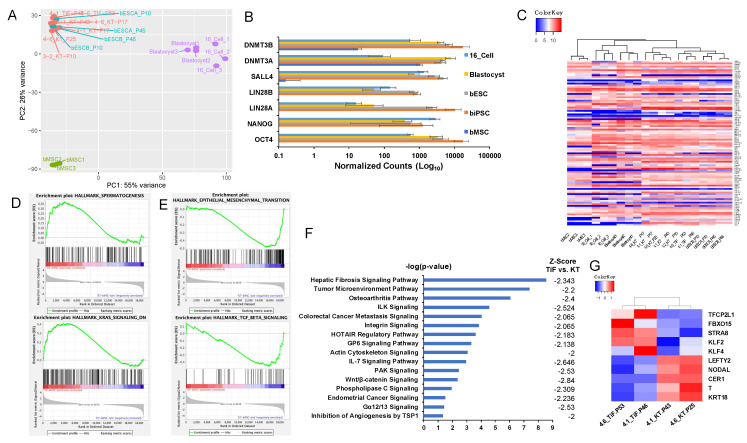
Transcription analysis of biPSCs. (**A**): PCA analysis of RNA-seq data from biPSCs in KT and TiF media, bESCs, and bovine embryos at the 16-cell and blastocyst stages. (**B**): Comparison of pluripotent gene expression between biPSCs, bESCs, bovine embryos, and bMSCs based on RNA-seq data. (**C**): Heatmap2 clustering analysis on 97 bovine pluripotent genes detected from RNA-seq data. (**D**): Biological processes enriched in naïve-like biPSCs by GSEA analysis. (**E**): Biological processes enriched in primed-like biPSCs by GSEA analysis. (**F**): Significantly inhibited signaling pathways in naïve-like biPSCs (in TiF medium) compared with primed-like biPSCs (in KT medium). (**G**): Heatmap2 analysis on naïve- and primed- markers for biPSCs in TiF and KT media based on RNA-seq data.

We analyzed the global transcriptome profiles of the biPSC lines, and compared them with the published data for the bovine embryos at the 16-cell and blastocyst stages [8,56], and bovine ESCs (bESCA/B from P10 to P46) [8] (GSE180931). Principle Component Analysis (PCA) revealed that the three lines of biPSCs in KT medium at different passages (4-1_KT-P17 and P43, 4-6_KT-P17 and P25, and 3-2_KT-P10) and two lines of biPSCs in TiF medium (4-1_TiF-P46, 4-6_TiF-P53) were clustered together with bovine ESCs (Figure 4A). The biPSCs expressed pluripotent genes comparable to the bovine embryos and ESCs, including OCT4, NANOG, LIN28A/B, SALL4, DNMT3A/ 3B (Figure 4B). Heatmap analysis for 4981 differentially expressed genes (DEGs) from bMSCs (absolute fold change (FC) >5, false discovery rate (FDR) <0.05) also revealed a high degree of similarity between the biPSCs and bovine embryos (Figure S2A, Table S1). Studies on human ESCs/iPSCs had identified a set of 169 pluripotent markers as a fingerprint for PSCs (StemCellDB) [57]. Data mining on our RNA-seq results identified 97 out of 100 annotated bovine genes orthologous to the StemCellDB fingerprint markers (Table S2). Heatmap analysis on the expression of these 97 bovine genes again clustered all the biPSCs together with the bovine embryos and ESCs (Figure 4C, Table S3).

To gain a deeper understanding of cell signal changes between the two types of biPSCs, we performed gene set enrichment analysis (GSEA) to identify significantly enriched biological state/process gene-sets with the *p*-value < 0.05 and FDR < 0.25 [58,59,60]. The spermatogenesis and genes downregulated by KRAS signaling were found significantly enriched in naïve-like biPSCs (Figure 4D, Table S4), whereas 26 biological states/processes were found highly enriched in primed-like biPSCs, with the top ten of these including the epithelial-to-mesenchymal transition (EMT), MYC-targets, interferon and inflammatory responses, oxidative phosphorylation, angiogenesis, and TGF-β signaling Figure 4E and Figure S2B, Table S5). We further used Ingenuity Pathway Analysis (IPA) [61] to analyze the canonical signaling difference between naïve-like and primed-like biPSCs. The IPA regulation z-score algorithm was used to identify activated or inhibited biological functions (absolute z-score ≥ 2). Sixteen pathways were found significantly inhibited in naïve-like biPSCs in TiF medium compared with primed-biPSCs in KT medium, including the hepatic fibrosis pathway, tumor microenvironment pathway, osteroarthritis pathway, etc. (Figure 4F, Table S6). Some of the signaling pathways correlated well with the biological processes identified in GSEA analysis, such as the colorectal cancer mestasis signaling vs. EMT, IL-7 signaling vs. interferon α/γ responses, osteroarthritis pathway vs. inflammatory response, etc. Therefore, inhibiting these pathways might be necessary to achieve naïve property from the primed-like biPSCs. Furthermore, comparing the expression of different pluripotent-stage markers between the two types of biPSCs showed that the naïve-like biPSCs had increased expression of naïve-pluripotent markers including TFCP2L1, KLF2/4 [62], FBXO15, and STRA8 [54,63], while the expression of primed-pluripotent markers including LEFTY2, NODAL, CER1, T [54,63,64,65], and KRT18 [64] were all downregulated (Figure 4G).

### 2.5. In Vivo Chimerism Capacity of Bovine iPSCs in Mouse Embryos

**Figure 5:**
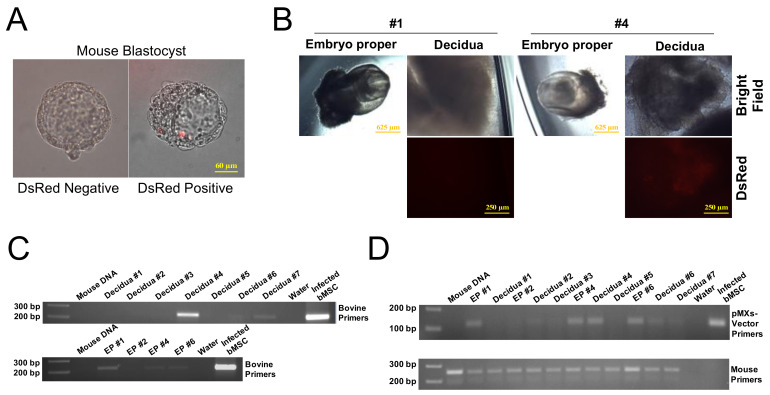
Mouse embryo chimerism analysis of biPSCs. (**A**): DsRed-biPSCs incorporation of mouse blastocyst (right) at 24 h after embryo aggregation. Bar = 60 µm. (**B**): DsRed-biPSCs contribution to the decidua of #4 (right) but not #1 (left) E8.5 mouse embryo. Bar = 625 or 250 µm as indicated. (**C**): PCR analysis of DsRed-biPSC contribution in E8.5 mouse decidual tissue (upper panel) and embryo proper (EP, lower panel) using bovine-specific primers. (**D**): PCR analysis of DsRed-biPSC contribution on E8.5 embryos using pMXs-vector primers (upper panel). PCR using mouse-specific primers (lower panel) served as reference.

Using the naïve-like biPSCs, we derived two biPSC lines with either constitutive expression of pMXs-DsRed or with Doxicyclin (Dox) inducible FUW-TetO-eGFP (DsRed- or GFP-Dox-iPSCs). While the vast majority of the infected biPSC colonies rapidly silenced DsRed expression within two to three days, which was consistent with the observed transgene silencing property of these cells, we did observe several colonies with faint but consistent DsRed expression and were able to expanded them in TiF medium (Figure S2). In order to evaluate the in vivo chimerism capacity of the biPSCs, we performed an aggregation experiment and co-cultivated early mouse morula (8-cell stage) with the DsRed-biPSCs (P51). At 24 h after aggregation, we fixed six mouse embryos, which developed into early blastocysts, and we were able to detect the presence of red fluorescence in one blastocyst (Figure 5A). The remaining blastocysts were then transferred into pseudopregnant female mice for further development. At E8.5, we recovered seven decidua with four containing well-developed embryos (#1, #2, #4, #6), whereas the other three were empty decidua likely due to embryo displasia. We did not find red fluorescence in mouse embryo proper, but observed DsRed in the decidual tissue harboring embryo #4 (Figure 5B). This indicates that biPSCs were incorporated into trophoblasts or extraembryonic mesoderm leading to subsequent development into chorion tissue. It is possible that expression of DsRed in biPSCs might be too faint to detect, or subject to retroviral silencing after incorporation into ICM of early mouse embryos. To further verify biPSC chimerism, we extracted genomic DNA from mouse embryo proper and decidual tissue separately, and performed PCR using bovine-specific primers [66] to amplify bovine 1.715 satellite DNA. We used genomic DNA isolated from mouse uterus and water as negative controls, and transgene infected bMSCs as positive control. We detected strong bovine-specific PCR product in the decidual DNA of embryo #4, along with weak, but detectable PCR product in the decidua of embryos #6 and 7 (Figure 5C, upper panel). Interestingly, PCR also detected positive band in the embryo propers #1, 4, and 6 (Figure 5C, lower panel). The presence of biPSC DNA in the mouse embryo propers and decidual tissues was further confirmed by PCR using pMXs-vector specific prime pair (Figure 5D). We therefore concluded that the biPSCs are capable of contributing to mouse embryonic and extra-embryonic tissues.

### 2.6. Efficiency of biPSCs as Donors for Somatic Cell Nuclear Transfer (SCNT)

**Figure 6:**
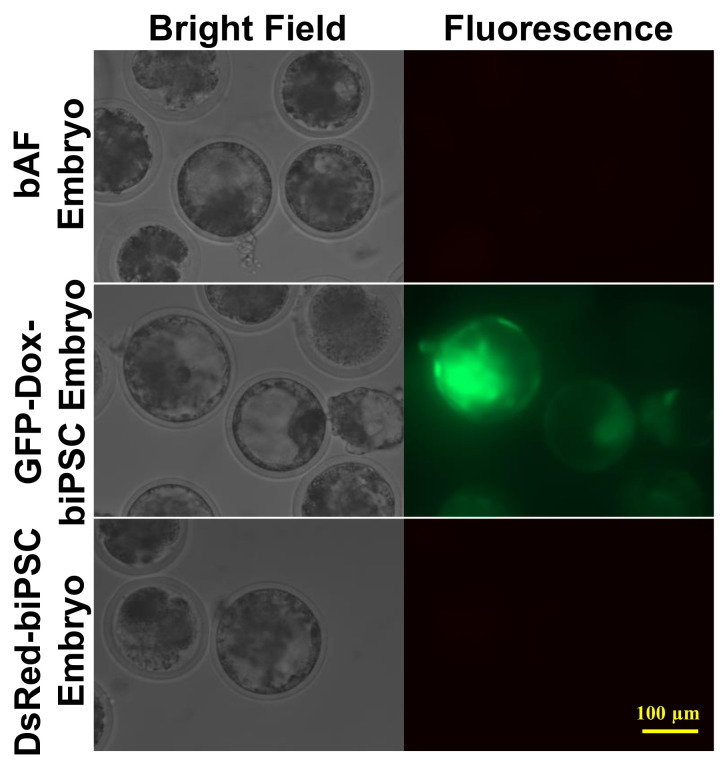
Blastocysts cloned from biPSCs. Blastocysts using bAF as nuclear donors (**upper panel**); GFP-Dox-biPSC as nuclear donors (**middle panel**), Dox was added into the embryo culture medium 6 days after activation of cloned embryos to induce the GFP expression; and DsRed-biPSC as nuclear donors (**lower panel**). Bar = 100 µm.

As the capacities of single cell colonization and infinite self-renewal make naïve-PSCs amenable for genetic modification and to serve as ideal nuclear donors for cloning, we next sought to determine the efficiency of biPSCs in bovine NT experiments. Both late passage DsRed-biPSCs (P55) and GFP-Dox-biPSCs (P56) were used as nuclear donors to generate cloned embryos. We observed no significant difference (*p* > 0.05) either in fusion or cleavage rates among two biPSC groups and a control group using bovine adult blastocysts (bAFs) as nuclear donors (Table 1). The two lines of biPSCs supported cloned blastocyst development at efficiencies of 24.7% (DsRed-biPSC) and 24.5% (GFP-Dox-biPSC), respectively (Table 1). There was no significant difference in blastocyst rates between the two biPSC lines and between biPSC and the control groups. When Dox was added on day six after embryo activation, we observed clear GFP expression in the cloned GFP-Dox-biPSC blastocysts (Figure 6). However, no red fluorescence was detected in blastocysts derived from the DsRed-biPSCs, although we could observe expression of red fluorescence in biPSC colonies cultured prior to SCNT. This is similar to what we had observed in the mouse embryo aggregation experiment (Figure 5B).

## 3. Discussion

The generation of bovine iPSCs capable of long-term self-renewal and without transgene activation has been extremely challenging [24]. In this study, using a combination of seven factors (K_d_OSKMLN), and the reprogramming medium containing inhibitors to WNT (IWR1) and H3K79 methyltransferase Dot1L (iDot1L), we report the induction of primed-like biPSCs, which do not rely on the continuous exogenous transgene activity for self-renewal. We further developed TiF medium to convert the primed-like biPSCs to naïve-like biPSCs capable of single cell colonization. The primed and naïve-like biPSCs are both capable of propagation for at least 60 and 70 passages in the laboratory, respectively. We further demonstrated that these cells can differentiate into cells of the three-germ layers in vitro, and found that the naïve-biPSCs can incorporate into both mouse embryonic and extra-embryonic tissues in vivo. At high passage numbers (>P55), these biPSCs served as nuclear donor for NT experiment, and gave an average of 24.6% blastocyst development rate, which is comparable to the early-passage adult fibroblast donors. The blastocyst rate from high passage biPSCs here appears higher than the reported blastocyst rates using early passage bESCs as donors (10–21.2%) [8,25], although variations on the experimental system needs to be considered between different studies. Moving forward, it would be interesting to compare early passage (P10-20) biPSCs with the late passages in cloning experiment to determine the impact of the long-term propagation of biPSCs to NT-blastocyst development.

H3K9me3 modification in somatic cells represents an obstacle for iPSC generation. In mice, overexpression of H3K9me3 demethylase Kdm4b or suppression of H3K9me3 methyltransferases Setdb1, Suv39h1, or Suv39h2 in reprogramming significantly improved iPSC induction efficiency [35]. Similarly, inhibition of SUV39H1, SUV39H2, or both in human somatic cells markedly increased the generation of human iPSC colonies [36,37]. Mechanistically, in human fibroblasts, H3K9me3 uniquely marks the heterochromatin regions, which blocks the binding of these regions by the OSKM transcription factors, therefore preventing the activation of these regions and impeding reprogramming process [37]. Thus, efficient removal of H3K9me3 in somatic cells is essential for the generation of completely reprogrammed iPSCs across species. We confirmed here that in bovine cells the KDM4A inhibits H3K9me3 but not H3K9me2 level, and reported the establishment of biPSCs using the six reprogramming factors plus KDM4A. It would be interesting to further investigate the heterochromatin regions marked by H3K9me3 in bovine cells, to identify essential DNA elements to be activated for the establishment of bovine PSCs.

During preparation of this manuscript, two studies reported the generation of biPSCs capable of long-term passage and with extended differentiation capacity. One study used eight factors (OSKMLN plus RARG and LRH1) to reprogram bovine fetal fibroblasts, and cultured biPSCs in mTeSR medium containing WNT inhibitor XAV939 (or IWR1), CHIR99021, Lck/Src inhibitor WH-4–023 or A419259, Vitamin C, ACTIVIN A, and LIF (bEPSCM medium) [25]. The other study used the same reprogramming system and cultured biPSCs in N2B27-based medium supplemented with KSR, LIF, CHIR99021, (S)-(+)-dimethindene maleate, and minocycline hydrochloride (LCDM medium) [67]. All reports including ours here used CHIR99021 and LIF. Also, similar to as reported previously [8], both the bEPSCM and TiF media contain an WNT inhibitor such as IWR1 to suppress bovine PSC differentiation. One difference is that our TiF medium contains the MEK1 inhibitor PD0325901, which induce naïve-state PSCs by suppressing the activation of downstream ERK1/2 signaling [44,45]. However, in the other report, inhibiting MEK by PD0325901 caused death of biPSCs [25]. Therefore, bovine PSCs with extended differentiation capacity for both embryos and trophectoderm could be cultivated in different medium conditions, including the TiF reported here that contains 2i/LIF components. One of our next steps will be to test the capacity of these biPSCs for in vivo chimera-generation in bovine embryos.

Although we successfully induced biPSCs from bovine somatic cells using this reprogramming system, the current reprogramming efficiency as measured by the number of AP-positive colonies over total starting reprogrammed cells was only around 0.05% (data not shown). A similar reprogramming efficiency (0.1%) was reported by the other group using PiggyBac system [25]. Another future task for us is to further optimize the reprogramming system, including using the powerful OCT4-MYC fusion protein strategy as reported previously [68], to further improve the reprogramming efficiency as well as to shorten the timing of biPSC induction.

The generation of completely reprogrammed biPSCs will provide invaluable PSC sources to facilitate both the basic research to understand cattle embryonic cell development and the applied studies to screen for genetic traits to improve reproduction, dairy/beef quality and productivity, and disease-resistance in cattle. Overall, we had developed a reprogramming system with K_d_OSKMLN factors that can be used to establish long-term passaged, transgene-silenced biPSCs. These important breakthroughs will greatly facilitate the establishment of *bona fide* bovine PSCs.

## 4. Materials and Methods

### 4.1. Chemicals, DNA Constructs, and Primary Bovine Cells

The DOT1L inhibitor EPZ004777 (iDOT1L) was purchased from AOBIOUS Inc. (Gloucester, MA, USA). WNT inhibitor IWR1, GSK-3 inhibitor CHIR-99021, MEK inhibitor PD0325901, ROCK inhibitor Y-27632 (ROCKi), and caspase inhibitor Z-VAD-FMK were purchased from Selleckchem (Houston, TX, USA). Forskolin was purchased from Fisher Scientific (Pittsburg, PA, USA). Human reprogramming factors OCT4, SOX2, KLF4, MYC, NANOG, LIN28A, and KDM4A were used for bovine somatic cell reprogramming. The constructs pMXs-OCT4, FUW-TetO-eGFP, and FUW-m2rtTA were purchased from Addgene (Cambridge, MA, USA). Construction of the polycistronic vector pMXs-KLF4, MYC, and SOX2 (KMS) [68] and pMXs-LIN28A and NANOG (LN) [30] were described previously. Human KDM4A was cloned into pMXs-vector similarly to as previously described in [30]. Primary bovine umbilical cord-derived mesenchymal stem cells (bMSCs) were collected from Wharton’s jelly part of the placenta of a newborn male Holstein calf, and were maintained with low serum MSC medium (ATCC, Manassas, VA, USA).

### 4.2. Reprogramming of bMSCs

For viral packaging, pMXs constructs were co-transfected into HEK293T cells with PUMVC and pCMV-VSV-G packaging plasmids, while FUW constructs were transfected with psPAX2 and pCMV-VSV-G plasmids (all from Addgene) using Fugene 6 (Promega, Madison, WI, USA) according to the protocol provided from the Addgene website. Virus-containing supernatant was harvested at 48 and 72 h post-transfection and filtered through 0.8 μm filters. Viral aliquots were stored at −70 °C until use. For reprogramming, on day minus one (−1), bMSCs were plated onto six-well tissue culture plates at a density of 5 × 10^5^ cells/plate. On days 0 and 1, retrovirus carrying K_d_OSKMLN were added with 10 μg/mL polybrene (AmericanBIO, Natick, MA, USA). On day 2, the cells were maintained in a 1:1 mix of MSC medium and knockout serum replacement (KSR) medium, which contains 20% KSR in DMEM/F12, supplemented with 1 × NEAA, 1 × Glutamax, and 0.5 × penicillin and streptomycin, 12 ng/mL human bFGF (all from Thermo Fisher Scientific, Waltham, MA, USA), and 1 × β-mercaptoethanol (Merck Millipore, Billierica, MA, USA). The infected cells on day 5 were passaged onto mitomycin C treated MEF feeders in the presence of 10 μM ROCKi. On day 20, the medium was changed to a 1:1 mix of KSR medium and the FAC medium, which consists of a 1:1 mix of DMEM/F12 and neutral basal medium supplemented with 1 × B27, 0.5 × N2, 1 × NEAA, 1 × Glutamax, 0.5 × penicillin and streptomycin, 1% KSR, and 0.05 μg/mL BSA. The 1:1 mix of KSR and FAC media is further supplemented with 12 ng/mL human bFGF (all from Thermo Fisher Scientific), 1× β-mercaptoethanol (Merck Millipore, Billierica, MA, USA), CHIR-99021 (3 μM), and Activin A (5 μg/mL). To inhibit the cell apoptosis during the viral infection and reprogramming, Z-VAD-FMK (20 μM) was supplemented in the medium during day 0 up to day 20. iDOT1L (3.3 μM) was applied in the media all the time, and IWR1 (10 μM) was added in reprogramming media since day 12. Starting from day 28, the biPSCs were maintained in a 1:1 mix of mTeSR-plus (STEMCELL Technologies, Cambridge, MA, USA) and KSR media supplemented with 1000 U/mL human LIF (Merck Millipore), 10 μM IWR1, 3.3 μM iDOT1L (KT medium) and passaged with collagenase. For some picked colonies, TiF medium was applied for reprogramming cells since passage 35, and passaged with trypsin or Tryple Express (Thermo Fisher Scientific). The TiF medium includes mTeSR plus medium, 1 μM PD0325901, 3 μM CHIR-99021, 1000 U/mL human LIF (Merck Millipore), 2.5 μM IWR1, 3.3 μM iDOT1L, and 10 μM Forskolin.

### 4.3. Quantitative Reverse Transcription-PCR (qRT-PCR) Analysis

Total RNAs were isolated from parental bMSCs, biPSCs, or differentiated cells with RNeasy mini kits (Qiagen, Hilden, Germany). Genomic DNAs were removed by DNase I (Qiagen) incubation. A total of 0.5 μg RNAs were then reverse transcribed into cDNA using iScript reverse transcription supermix (Bio-Rad Laboratories, Hercules, CA, USA). qRT-PCR reactions were performed with SYBR Green supermix (Bimake, Houston, TX, USA) using the ABI 7500 Fast platform (Thermo Fisher Scientific). GAPDH was used as the housekeeping gene for gene expression normalization. Data were processed with the software associated with ABI 7500.

### 4.4. Embryoid Body (EB) Differentiation

EB formation experiments were carried out with bovine iPSC lines. When growing to 70–80% confluency with mainly middle-size colonies, the cells were treated with freshly prepared 1 mg/mL collagenase for 30 min and removed from the plate by pipetting. After three washes with DMEM/F12, the cells were then plated onto low-adhesive petri dishes in EB formation medium on day 0 (KSR medium without bFGF). Half of the medium were changed to DMEM with 10% FBS every other day. EBs were treated by 0.05% Trypsin (Thermo Fisher Scientific) on day 5 and plated onto gelatin-coated plates. EBs at day 5 and day 14 were harvested for RNA isolation and gene expression analysis. The cells were subjected to immunofluorescence staining on days 12–14.

### 4.5. Immunostaining

For immunofluorescence, the cells were first fixed in 4% PFA for 15 min at room temperature. Following fixation, the cells were treated with 0.5% Triton X-100 in PBS for 15 min at room temperature for cell membrane permeabilization. After blocking with goat serum (Cell Signaling Technology, Danvers, MA, USA), the cells were incubated in goat serum containing primary antibodies for 2 h at 37 °C, followed by secondary antibodies at room temperature for 1 h. Cells were counter-stained with DAPI and imaged under a Nikon fluorescence microscope. Primary antibodies including rabbit anti-OCT4 (Santa Crutz, CA, USA), rabbit anti-SOX2 (Merck Millipore), and mouse anti-NANOG (Merck Millipore) were used at 1:100 dilution while mouse anti-AFP (1:50; Cloud-clone Corp., TX, USA), rabbit anti-TUJ1 (1:500) and mouse anti-SMA (1:400) (Thermo Fisher Scientific) were diluted variously according to their manufacturer’s instructions. Alexa Fluor 488 or 594 conjugated goat anti-rabbit or goat anti-mouse secondary antibody (Cell Signaling Technology) were used in 1:1000 dilution. For cell surface marker staining, the cells in different reprogramming conditions were stained with NL557-conjugated TRA-1-60 (1:100), SSEA-3 (1:50; R&D Systems, Minneapolis, MN, USA) and Alexa Fluor 594 conjugated SSEA-4 (1:100; BioLegend, San Diego, CA, USA) according to the manufacturer’s protocol.

### 4.6. RNA-seq Data Analysis

Total RNA was isolated from reprogrammed cells with different treatments using RNeasy Mini kit (Qiagen). The quality of total RNA was examined by Nanodrop, Agarose Gel Electrophoresis, and the Aglient 2100 bioanalyzer. rRNA was then removed by using Ribo-Zero-rRNA Removal kit (Epicentre, Madison, WI, USA). First, the mRNA was fragmented randomly by adding fragmentation buffer, then the cDNA was synthesized by using mRNA template and random hexamers primer, after which a custom second-strand synthesis buffer (Illumina), dNTPs, RNase H, and DNA polymerase I were added to initiate the second-strand synthesis. Second, after a series of terminal repair, A ligation and sequencing adaptor ligation, the double-stranded cDNA library was completed through size selection and PCR enrichment. Finally, sequencing libraries were quantified by using Agilent 2100 bioanalyzer and then fed into Illumina sequencers.

The RNA-seq data analysis was conducted at usegalaxy.org. Sequencing adapters and reads with low quality were trimmed using Cutadapt before mapping. The quality of reads after filtering was examined using fastQC. For mapping, bovine genomic sequence and RefSeq gene coordinate (ARS-UCD1.2/bosTau9) were downloaded from the UCSC genome browser. All filtered reads were aligned to bovine reference genome by RNA STAR (Galaxy Version 2.7.8a) with default parameters. The number of reads per gene was counted by feature Counts (Galaxy Version 2.0.1). Differentially expressed genes between different samples were identified using default parameters in DESeq2 (Galaxy Version 2.11.40.6 + galaxy1), which generated a principal component analysis plot and the heatmap of the sample-to-sample distance matrix. The most differentially expressed genes (adjusted *p*-value < 0.05) were extracted from DESeq2 results with an absolute fold change (FC) >5. The normalized counts for those differentially expressed genes and the Z-score of the counts were calculated on the galaxy platform and exhibited as heatmaps by Heatmap2 (Galaxy Version 3.0.1). The normalized counts for naïve- and primed-like biPSC samples were subjected to GSEA analysis (gsea-msigdb.org), with the log2FC values used for IPA analysis (Qiagen).

### 4.7. Karyotyping

Karyotyping was carried out on biPSCs at different passages. biPSCs were first incubated with 10 μg/mL colcemid (Sigma-Aldrich, St. Louis, MO, USA) at 37 °C for 2 h, following which the cells were harvested by trypsinization. The cells were then incubated in hypotonic solution (0.56% KCl solution) for 15 min at 37 °C. After three times washing in the fixative solution (methanol/glacial acetic acid 3:1), the cells were dropped onto wet and ice-cold glass slides. Giemsa (Sigma-Aldrich) at 1:20 dilution was applied onto the dried slides for staining. The nuclei were visualized with an Olympus microscope under a 100× oil objective lens.

### 4.8. Bisulfite Sequencing

For bisulfite sequencing, genomic DNAs were extracted and bisulfite converted using the EpiTeck Bilsulfite Kit (Qiagen). Bovine OCT4 and NANOG proximal promoter regions were amplified using PCR primers previously reported [10], and OCT4 distal primers were designed on MethPrimer [69]. The sequences of the primers are as follows. For OCT4 proximal promoter region primers, forward primer: 5′-GTTTGGAGAGGGGTTTTGAAGAATGTGTAG-3′, and reverse primer: 5′-ATCCCACCCACTAACCTTAACCTCTAAC-3′. For OCT4 distal enhancer region primers, forward primer: 5′-TGTTTGGAGAATTTTATGGATAGAG-3′, and reverse primer: 5′-CCAATTAAATCATCAAACCTAACTC-3′. For NANOG proximal promoter region primers, forward primer: 5′- TAGGTGGTTATAGGAGATGTATTTTTGATT-3′, and reverse primer: 5′-TTATAAATAAAACTCAACCATACTTAACCC-3′. PCR were performed with Hot Start 2 × Master Mix (Thermo Fisher Scientific) and cloned using the In-Fusion HD Cloning System into pIRES2-DsRed vector (Clontech) digested by BglII and EcoRI (New England Biolabs, Ipswich, MA, USA). Clones were picked, cultured in 6 mL LB medium with antibiotics overnight, and plasmid DNAs were extracted using a Qiaprep Mini Kit (Qiagen) and were subject to regular Sanger DNA sequencing. The results were then analyzed using the BiQ Analyzer [70].

### 4.9. Aggregation to Generate Chimeric Embryos

Chimeric embryos were generated by biPSC <-> mouse embryo aggregation as described previously [71]. Briefly, E2.5 morulae were isolated from CD-1 females (Charles River). Zona pellucidae were removed by brief exposure to acidic Tyrode’s solution (Sigma T1788) followed by several washes in KSOM embryo medium (Millipore MR-101-D). Zona-free embryos were placed individually in micro-wells with KSOM embryo medium in an aggregation plate covered with light mineral oil (Fisher Cat#01211). Bovine iPSCs were fed with fresh culture media 2 h before aggregation. Cells were washed 2× with PBS, dislodged from the plates by brief exposure to 0.05% Trypsin (Sigma SM-2003-C) to obtain clumps of 8 to 12 cells. Two clumps of cells were then placed with a zona-free embryo in the micro-well and co-cultivated together in an 37 °C incubator with 6% CO_2_, 5% O_2_ and 89% N_2_. After an overnight incubation, biPSC <-> mouse embryo aggregates that did not develop into blastocyst were discarded and 20 to 25 blastocysts from each line were then transferred into pseudopregnant females for subsequent development. Embryos at various stages of gestation were harvested for analysis. For genotyping in mouse chimeras, genomic DNA was isolated from mouse embryos or extraembryonic tissue using DNeasy Blood & Tissue Kit (Qiagen). A total of 100 ng of genomic DNA was used for each PCR reaction with primers specific for mouse (forward primer: 5′-TGTGGGCAAAGAGGCTTCAT-3′, reverse primer: 5′-CAAAGCTGACTTAGCCTCAG-3′), bovine (forward primer: 5′-TGAGGCATGGA- ACTCCGCTT-3′, reverse primer: 5′- GGTGGTTCCACATTCCGTAGGAC-3′), and the pMXs-vector (forward primer: 5′-CCGGTCGCTACCATTACCAG-3′, reverse primer: 5′-CGGCCGCTCGAGTTTAAATA-3′) sequences using Hot Start 2 × Master Mix.

### 4.10. Nuclear Transfer Using biPSCs as Nuclear Donors

The biPSCs were cultured in a 6-well plate at 37 °C, 5% CO_2_ in a humidified atmosphere. After culture for 4–5 days, the biPSCs forming typical compact colonies were used as nuclear donors for NT. Three hours before NT, biPSC culture medium was refreshed. Thirty minutes before NT, the biPSCs in one well of the 6-well plate were digested with 0.3 mL of Tryple Express (Cat. 12605-010, Gibco). The Tryple Express was neutralized by 5-fold dilution in the biPSC culture medium 8 min after digestion. The mixture was then centrifuged at 300× *g* for 5 min and the supernatant removed. The cell pellets were resuspended in 0.1 mL of the biPSC culture medium. The resuspended biPSCs were stored at room temperature (20–22 °C) before use. The bovine adult fibroblasts (bAFs) at passage 8–10 were grown to 80–90% confluence and used as nuclear donor cells in the control group after 24 h of serum starvation (0.5% FBS in DMEM).

Bovine SCNT was performed as described by Fan et al. for goats [72], with modifications wherein an aspiration technique was used for oocyte recovery instead of a slicing technique and bovine oocyte maturation and culture media instead of caprine media. The bovine oocyte maturation medium consists of TCM-199 supplemented with 10% FBS, 5 μg/mL luteinizing hormone, 0.5 μg/mL follicle stimulating hormone, and 100 U/mL penicillin/streptomycin. The cloned embryos were in vitro cultured in bovine SOFaa medium with 5% FBS [73] for 7 days after activation. On day 6, the cloned embryos derived from GFP-Dox-biPSCs were transferred into a prewarmed SOF-Dox drop, which consists of the SOF medium supplemented with 1 µg/mL of doxycycline, to induce the GFP expression. The in vitro development of cloned embryos was observed under a stereo microscope and GFP or DsRed expression detected by a fluorescent microscope (Observer Z1, Zeiss).

### 4.11. Statistial Analysis

One way–ANOVA with Tukey’s multiple comparison post hoc test or Student’s t-test was used for data analysis. The figures were presented as mean ± standard deviation (sd). A *p*-value < 0.05(*) or 0.01(**) was considered statistically significant. For NT experiment, the data from fusion and development of cloned embryos were analyzed using square arcsine transformation, followed by one-way analysis of variance (ANOVA). A *p* value of < 0.05 for effects of factors was considered significant. A post hoc procedure with least significant difference (LSD) tests was used for multiple comparisons between groups. Means from differential staining were compared by one-way ANOVA (Jamovi). All the rest of the data were analyzed with SPSS platform.

## 5. Conclusions

We report here the successful generation of bovine iPSCs from somatic cells using the H3K9me3 demethylase KDM4A plus OSKMLN reprogramming factors. The established biPSCs exhibited silenced transgene expression with prolonged self-renewal capacity, high elevation of endogenous pluripotent factors, and displayed primed- or naïve-like pluripotent properties upon culturing in different medium conditions. The naïve-like biPSCs in high passage numbers contributed to both embryonic and extra-embryonic sections of mouse embryos, and could achieve a bovine blastocyst rate of at least 24.5% as nuclear donors in NT experiment. The established primed- and naïve-like biPSCs can serve as great resources for bovine embryology and biotechnology studies. Our results represent a significant advancement for the establishment of *bona fide* farm animal PSCs, especially from cattle.

## Figures and Tables

**Table 1:** Fusion Rates and In Vitro Developmental Capacities of Cloned Bovine Embryos Generated Using biPSCs as Nuclear Donors.

Donor Cells	No. of Oocytes	No. of Fused (mean ± sd%)	No. of Reconstructed Embryos	No. of Cleaved Embryos (mean ± sd%)	No. of Blastocysts (mean ± sd%) *
DsRed-biPSC	194	170 (88.4 ± 5.4) ^a^	123	89 (70.5 ± 17.0) ^a^	22 (27.0 ± 9.0) ^a^
GFP-Dox-biPSC	206	183 (88.9 ± 1.9) ^a^	131	98 (74.9 ± 10.8) ^a^	24 (25.2 ± 15.2) ^a^
bAF	188	172 (91.5 ± 0.5) ^a^	125	111 (88.6 ± 2.0) ^a^	45 (39.3 ± 10.7) ^a^

* Blastocyst rates were calculated from the number of cleaved embryos. ^a^ No significant difference (*p* > 0.05) were observed among 3 groups in the same column. The data were analyzed by one-way ANOVA, *n* = 4.

## Data Availability

The data presented in this study are openly available in GEO at [https://www.ncbi.nlm.nih.gov/geo/query/acc.cgi?acc=GSE180931]. Reference number [GSE180931].

## Supplementary Materials

The following are available online at https://www.mdpi.com/article/10.3390/ijms221910489/s1.
