# Supplementary material for: Establishment of Bovine-Induced Pluripotent Stem Cells
Source: Int J Mol Sci. 2021 Sep 28;22(19):10489. doi: 10.3390/ijms221910489 (PMC8508593; doi:10.3390/ijms221910489)
Supplement: Supplementary file 1 [file ijms-22-10489-s001.zip › Supplementary Files/Figure S1.pdf]

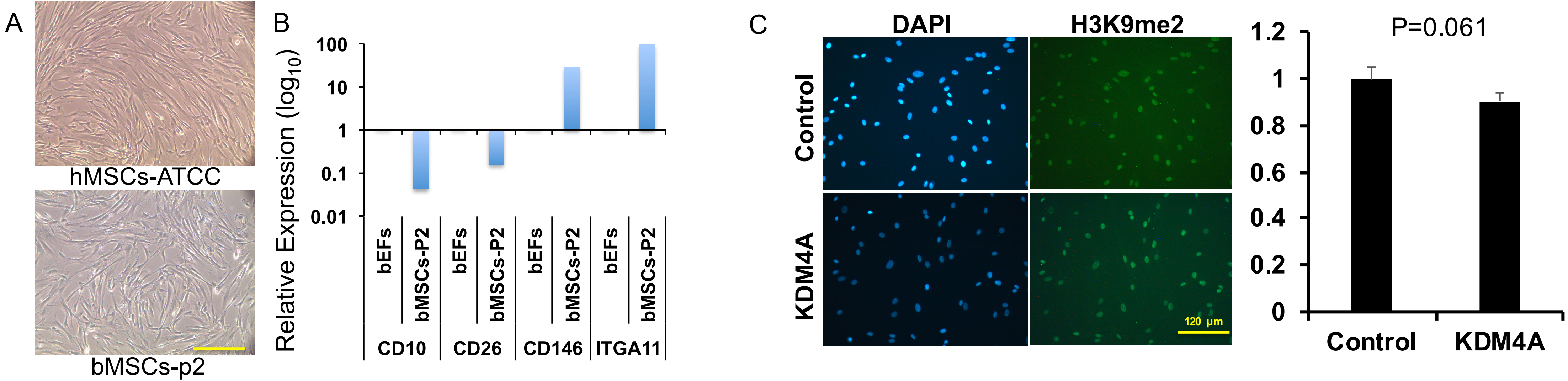

Figure S1: Generation of Primary Bovine MSCs. A: Pictures of primary human MSCs (hMSCs, from ATCC) and bovine MSCs (bMSCs) generated in house at passage 2 (p2). B: qPCR comparison for marker gene expression in bMSCs (CD146, ITGA11) and bovine embryonic fibroblasts (bEFs) (CD10, CD26). Values are normalized with GAPDH and relative to bEFs. C: Left Panel: Immunostaining of H3K9me2 expression in bMSCs infected with control vector or KDM4A. Bar = 120 μm. Right Panel: Relative fluorescence intensity for H3K9me2 in bMSCs. Bar=mean±sd, N=3. Student's t-test was used for data analysis.
