# Supplementary material for: Establishment of Bovine-Induced Pluripotent Stem Cells
Source: Int J Mol Sci. 2021 Sep 28;22(19):10489. doi: 10.3390/ijms221910489 (PMC8508593; doi:10.3390/ijms221910489)
Supplement: Supplementary file 1 [file ijms-22-10489-s001.zip › Supplementary Files/Figure S2.pdf]

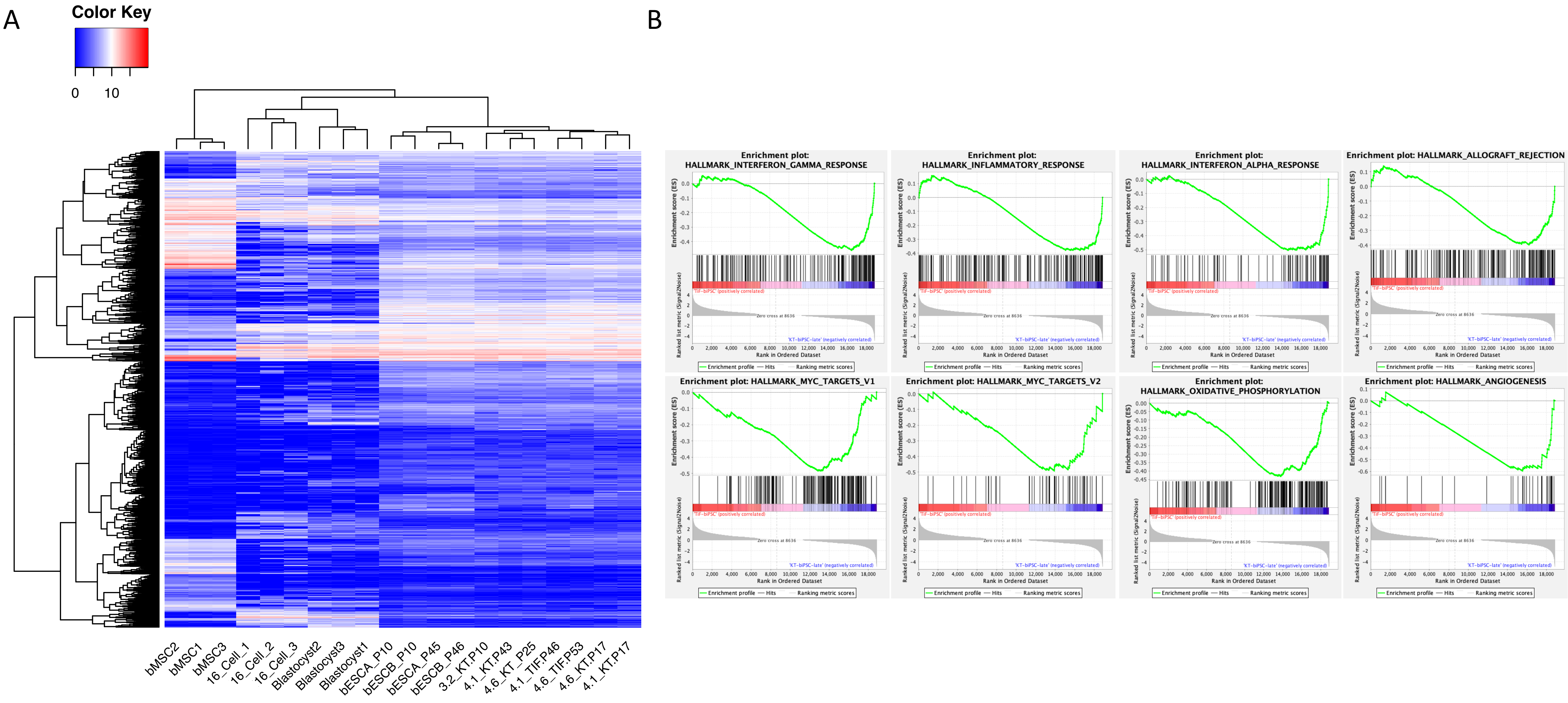

Figure S2: Transcription Analysis of biPSCs - Continued. A: Heatmap2 clustering analysis on 4,981 DEGs of different RNA-seq samples (FC > 5, FDR < 0.05). B: Additional significantly inhibited signaling pathways in naïve-like biPSCs (in TiF medium) compared with primed-like biPSCs (in KT medium).
