# Supplementary material for: Establishment of Bovine-Induced Pluripotent Stem Cells
Source: Int J Mol Sci. 2021 Sep 28;22(19):10489. doi: 10.3390/ijms221910489 (PMC8508593; doi:10.3390/ijms221910489)
Supplement: Supplementary file 1 [file ijms-22-10489-s001.zip › Supplementary Files/Figure S3.pdf]

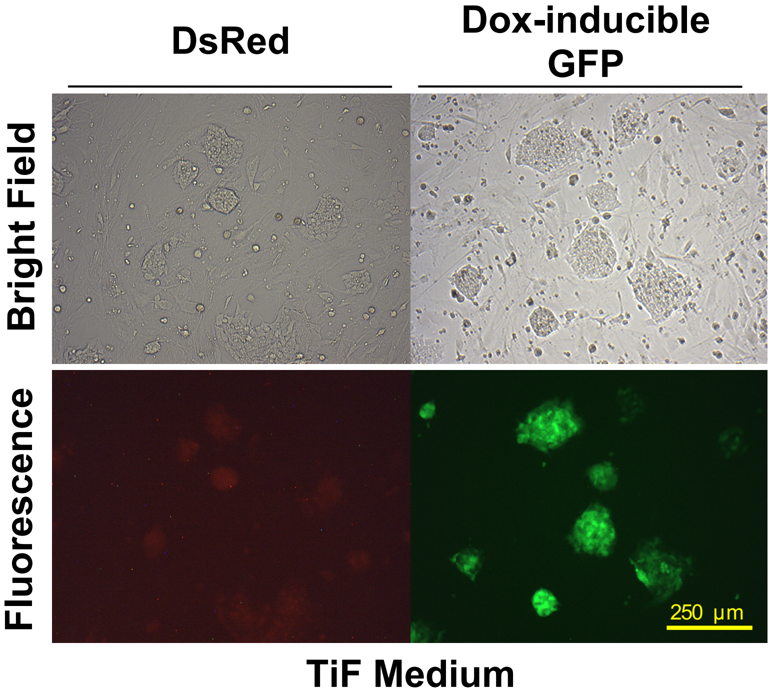

Figure S3: Generation of Fluorescent biPSCs. Left: Bright field and DsRed fluorescence of biPSC colonies. Right: Bright field and GFP fluorescence of biPSC colonies after Dox induction overnight. Bar=250 μm.
